# A scoping review on the association of early childhood caries and maternal gender inequality

**DOI:** 10.1186/s12903-023-03216-3

**Published:** 2023-07-26

**Authors:** Ivy Guofang Sun, Duangporn Duangthip, Charis Hiu-Kei Kwok, Chun Hung Chu, Yasmi O. Crystal, Robert J. Schroth, Carlos Alberto Feldens, Jorma I. Virtanen, Ola Barakat Al-Batayneh, Balgis Gaffar, Tshepiso Mfolo, Maha El Tantawi, Simin Z. Mohebbi, Hamideh Daryanavard, Morenike Oluwatoyin Folayan, Early Childhood Caries Advocacy Group (ECCAG)

**Affiliations:** 1 Faculty of Dentistry, The University of Hong Kong, Hong Kong SAR, China; 2 Faculty of Medicine, The University of Hong Kong, Hong Kong SAR, China; 3 College of Dentistry, New York University, New York, USA; 4 Dr. Gerald Niznick College of Dentistry, University of Manitoba, Manitoba, Canada; 5 Department of Paediatric Dentistry, Lutheran University of Brazil, Canoas, Brazil; 6 Faculty of Medicine, University of Bergen, Bergen, Norway; 7 Preventive Dentistry Department, Jordan University of Science and Technology, Irbid, Jordan; 8 Department of Preventive Dental Sciences, College of Dentistry, Imam Abdulrahman Bin Faisal University, Dammam, Saudi Arabia; 9 Department of Community Dentistry, University of Pretoria, Pretoria, South Africa; 10 Department of Paediatric Dentistry and Dental Public Health, Faculty of Dentistry, Alexandria University, Alexandria, Egypt; 11 Department of Community Oral Health, School of Dentistry, Tehran University of Medical Sciences, Tehran, Iran; 12 Dental Service Department, Dubai Academic Health Corporation, Dubai, United Arab Emirates; 13 Department of Child Dental Health, Obafemi Awolowo University, Ile-Ife, Nigeria

**Keywords:** Sex, Gender equality, Sustainable Development Goal, Early childhood caries, Dental caries, Preschool children

## Abstract

**Aim:**

The objective of this scoping review is to present current evidence regarding the association between early childhood caries (ECC) and maternal-related gender inequality.

**Methods:**

Two independent reviewers performed a comprehensive literature search using three databases: EMBASE, PubMed, and Web of Science. Literature published in English from 2012 to 2022 was included in the search and was restricted to only primary research by using the following key terms: "dental caries", "tooth decay", "gender", "sex", "preschool", "toddler," and "infant". The included studies were limited to those reporting an association between ECC and maternal aspects related to gender inequality. Titles and abstracts were screened, and irrelevant publications were excluded. The full text of the remaining papers was retrieved and used to perform the review. The critical appraisal of selected studies was guided by the Joanna Briggs Institute (JBI) Critical Appraisal Tools.

**Results:**

Among 1,103 studies from the three databases, 425 articles were identified based on publication years between 2012 and 2022. After full-text screening, five articles were included in the qualitative analysis for this review. No published study was found regarding a direct association between ECC and maternal gender inequality at the level of individuals. Five included studies reported on the association between ECC and potential maternal-gender-related inequality factors, including the mother’s education level (*n* = 4), employment status (*n* = 1), and age (*n* = 1). Regarding the quality of the included studies, out of five, two studies met all JBI criteria, while three partially met the criteria.

**Conclusions:**

Based on the findings of this scoping review, evidence demonstrating an association between gender inequality and ECC is currently limited.

## Introduction

Early childhood caries (ECC) is a non-communicable disease characterized by the presence of one or more decayed (non-cavitated or cavitated lesions), missing (due to caries), or filled surfaces in any primary tooth of a child under six years old (≤ 71 months) [1]. It is considered one of the most prevalent diseases in childhood. A review conducted in 2018 found that the mean ECC prevalence among children aged 36 to 71 months is estimated to be more than 50% globally [2]. ECC can cause infections, toothache, and abscesses, with a direct impact on the oral and general health of young children [3]. Severe ECC can impact children’s oral health-related quality of life [4]. Risk factors such as socioeconomic status (maternal education, family income, etc.), behaviour (early introduction of sucrose, frequent consumption of sugar, non-use of fluoridated toothpaste, practices of oral hygiene, etc.), and biological parameters are associated with the prevalence of ECC [5, 6].

Literature is emerging regarding the macro-social determinants of health, one of which may be cultures that perpetuate gender inequalities [7]. Gender norms perpetuate gender inequalities [8]. Restrictive gender norms and values shape social expectations regarding how individuals of a particular gender and age are expected to behave in a given social context [9]. It can affect all children but has been proven to affect girls disproportionately. More than 575 million girls live in countries in which inequitable gender norms contribute to violations of their rights [10]. In other parts of the world, gender norms and societal structures dictate that the flexibility and physical activity of women and girls are frequently controlled. This can be exacerbated by factors associated with income, household hierarchies, and roles [11].

**Fig. 1:**
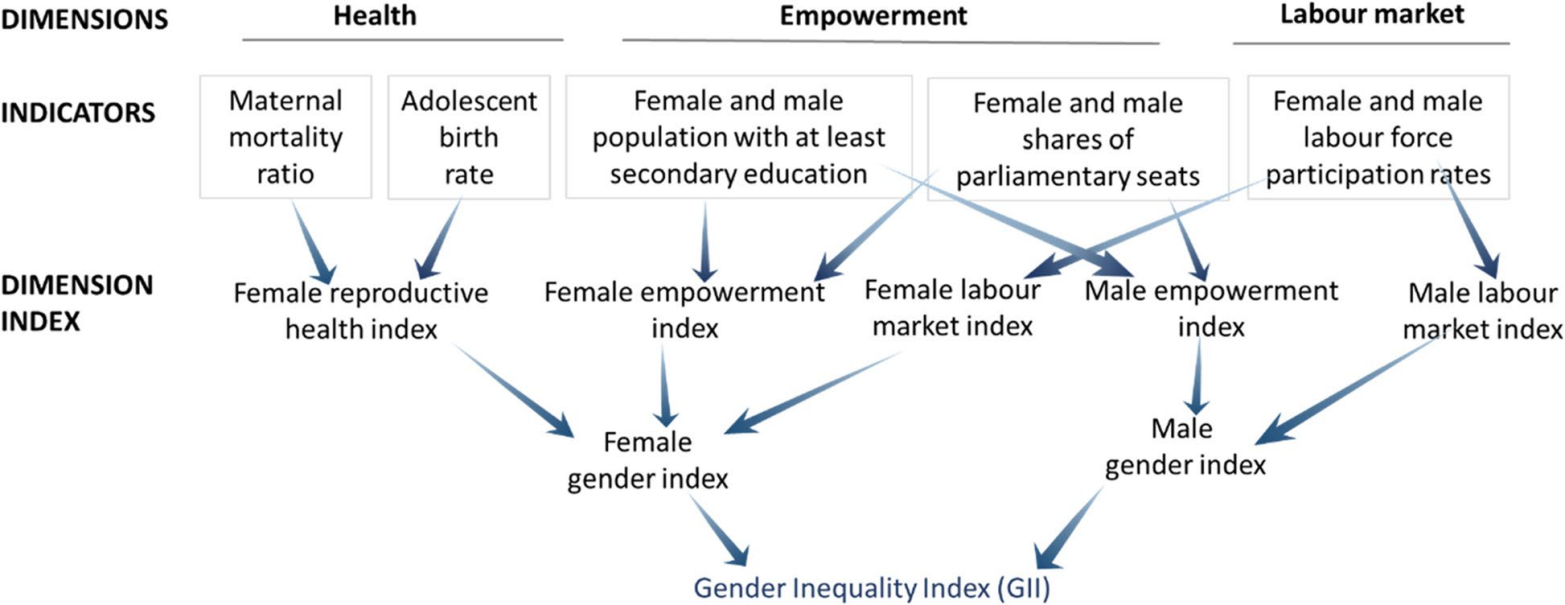
Gender inequality index (GII) (From United Nations Development Program [12])

Gender equality is recognized as a fundamental human right and is part of the goals set in the 2030 Agenda for Sustainable Development. The gender inequality index (GII) is a combined measure focused on the inequality in achievements between men and women in three main dimensions, namely empowerment, reproductive health, and the labour market (Fig. 1) [12]. The empowerment dimension is measured by the proportion of parliamentary seats held as well as levels of secondary and higher education attained by each gender. The maternal mortality ratio and the adolescent fertility rate can also be used to assess the health dimension. The labour dimension is measured by women's labour-force participation. The agenda emphasizes the empowerment of women and girls. Women, especially the underprivileged, bear most of the health consequences related to gender inequality [13]. Gender stereotypes can also cause health disparities between men and women [14]. A direct effect of gender norms on the risk of ECC may result from the differential access of girls and boys to the prevention of dental caries, including access to dental services or caries-prevention tools. Children may also be indirectly impacted by gender norms due to poor access of mothers or female caretakers to quality healthcare, thereby determining the utilization of healthcare services by preschool children [15]. Mothers' ability and rights to access healthcare are closely linked to their children's health and well-being, as mothers tend to conform to female gender roles as the main caretakers of their children [15].

Evidence for an association between gender inequality and health outcomes in children has been found [16, 17]; however, the effect of gender inequality on ECC is yet to be explored. The purpose of this review is to present current evidence regarding the association between early childhood caries (ECC) and maternal-related gender inequality.

## Methods

### Data sources and search strategy

This review was guided by two questions: "What is the existing evidence regarding the association between ECC and maternal gender inequalities?" and "What are potential maternal-related gender inequality factors (younger age, low education level, and unemployment of mothers) associated with ECC?".

The initial search was performed in March 2022 on three electronic databases: PubMed, EMBASE, and Web of Science. The search was conducted using the following key terms: "dental caries", "tooth decay", "gender", "sex", "preschool", "toddler," and "infant". Search terms were tailored to the specific requirements of each database. Search strategy for EMBASE: #1: *dental caries, #2: *sex, #3: *gender or “gender and sex”, #4: *preschool child, #5: *toddler, #6: *infant, #7: 1 and (2 or 3), #8: 1 and (4 or 5 or 6), #9: 7 and 8. Filter: 2012–2022; Search strategy for PubMed: ("dental caries" OR "tooth decay") AND (gender OR sex) AND (preschool OR toddler OR infant). Filters: from 2012–2022; Search Strategy for Web of Science: ("dental caries" OR "tooth decay") AND (gender OR sex) AND (preschool OR toddler OR infant). Filter: 2012–2022. The search was completed in April 2022. No protocol has been published for this review.

### Eligibility and selection

Literature obtained through database searches was exported to the reference-management software EndNote version X9; duplicates were removed using the "Find duplicates" function. The remaining duplicates that were not identified by the software were removed manually when encountered later in the review process. Screening of titles and abstracts was performed by two independent reviewers (IS and CK), guided by the eligibility criteria for this review. No authors or institutions were contacted to identify additional sources.

### Inclusion criteria

This review included only English-language publications related to ECC from January 2012 to April 2022. Studies included present the following information at the individual level:

A) Gender inequality and caries experience of children aged five years and below:
  Following the GII [12], studies of the association between gender inequality (including empowerment, female reproductive health, and the labour market) and ECC (caries prevalence or experience) in children aged five years (71 months) or below were included.
B) Potential factors related to gender inequality such as adolescent pregnancies, unemployed mothers, low education levels of mothers, and caries experience of children aged five years or below

### Exclusion criteria

As this review was intended to explore the association between ECC and gender inequalities with a focus on maternal factors following the GII, studies of the relationship between children's gender and ECC prevalence were excluded from this review. Literature published in languages other than English was also excluded. Papers with a sample population older than preschool age (5 years) were excluded. Because income level is not part of the index components for gender-equality measurements [18], the income level of the mother was also excluded.

### Data charting

Data extraction from the publications was done by two independent reviewers (IS and CK) during the publication screening and selection stage. The extracted information was compared, and if there were any doubts, a senior researcher (DD) was consulted and made the final decision. The following information was extracted from the publications including the following information: author, publication year, study location, study design, study sample size and age, study aim, data-collection methods, and main findings. The extracted information from each publication was then compiled and summarized in one table (Table 1). The GII and its ranks of the study countries are also added in Table 1 [19]. Critical appraisal of selected papers was guided by JBI critical appraisal tools.

**Table 1:** Summary of included articles (*n* = 5)

**Author (Publication year)**	**Location **[12] **(GII**^**a**^**, Rank)**	**Design**	**Sample num age**	**Aim**	**Data collection**	**Main findings**
***Age***						
Schüler et al. [20] (2018)	Germany (0.073, 19)	Cross-sectional	*N* = 128 3–4 years	Assess dental health in primary dentition of preterm infants and investigate mother-and-infant-related risk factors	Dental examinations & questionnaires & medical records	Preterm infants (PTI) whose mothers were > 25 years old at delivery had a lower risk of developing dental caries compared to PTI with younger mothers
***Education***						
Kato et al. [21] (2017)	Japan (0.083, 22)	Cross-sectional	*N* = 6,315 3 years	Examine the associations between parental occupations, levels of education, household income and the prevalence of dental caries in children aged 3 years old	Dental records & questionnaires	Compared with less than 13 years of maternal education (20%), mothers with 13–14 years (14%) and 15 or more years of education (12%) were inversely associated with the prevalence of dental caries in children
Shen et al. [22] (2020)	China (0.192, 48)	Longitudinal	*N* = 772 Mean age = 51 months	Assess socioeconomic inequalities in the increment of dental caries and growth among preschool children	Dental examinations & questionnaires	Mother’s education was negatively associated with increments of dmft
Sun [23] (2020)	China (0.192, 48)	Cross-sectional	*N* = 337 24–37 months	Determine if there is an association between postnatal depression and ECC	Dental examinations & questionnaires	A higher education level of mothers was associated with a higher possibility of their children having ECC experience
Al-Meedani et al. [24] (2016)	Saudi Arabia (0.247, 59)	Cross-sectional	*N* = 388 3–5 years	Determine the prevalence of dental caries and the associated social risk factors among preschool children	Dental examinations & questionnaires	Children’s caries prevalence was associated with maternal educational level. Children of mothers with doctorate/master’s degrees had lower caries prevalence (57%) than those with bachelor’s degrees (66%) and high school level or below (78%)
***Employment***						
Kato et al. [21] (2017)	Japan (0.083, 22)	Cross-sectional	*N* = 6315 3 years	Examine the associations between parental occupations, levels of education, household income and the prevalence of dental caries	Dental records & Questionnaires	Compared with having an unemployed mother (16%), having a mother who worked in professional and engineering (12%) or service (14%) was significantly inversely associated with the prevalence of dental caries

This table summarized all articles included in the final search

^a^Gender Inequality Index

## Results

**Fig. 2:**
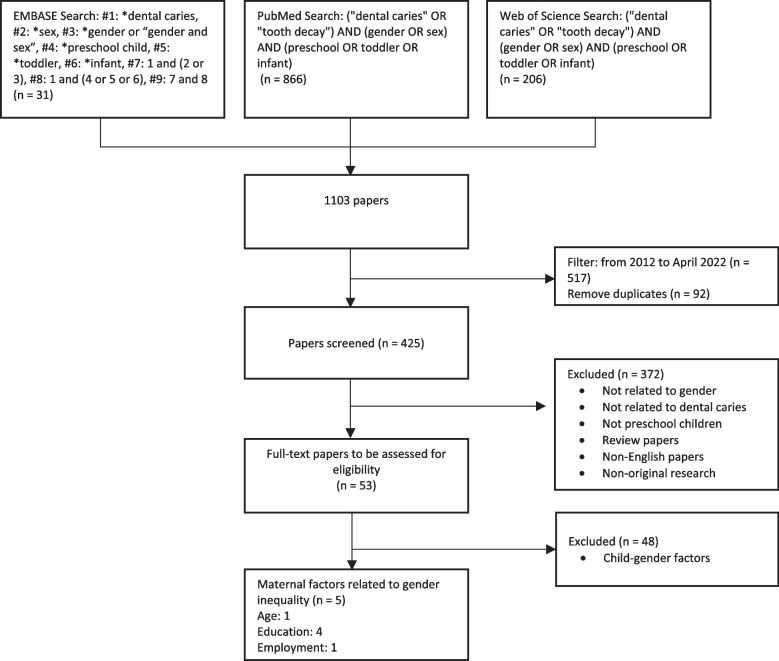
Flow chart of study selection process

The initial search using the specified search terms from the three databases (EMBASE, PubMed, and Web of Science) yielded 1,103 potentially relevant publications. A total of 517 papers published between January 2012 and April 2022 were identified to represent the current maternal gender inequality status. Ninety-two papers were removed as duplicates; the remaining 425 papers were then subjected to title and abstract screening. Following the specified inclusion and exclusion criteria, 372 papers were excluded. Thus, 53 papers were identified as eligible for full-text screening. Among them, 48 papers investigating the relationship between children's gender and ECC prevalence were excluded. A total of five papers regarding the association of potential gender-inequality-related factors such as younger age, unemployment, or low education level of mothers and ECC were included in the analysis for this review. None of the studies investigated a direct association between GII and caries experiences of children aged five years and below. The flow of publication identification is shown in Fig. 2

A summary of the results from the critical appraisal is presented in Tables 2 and 3, reflecting the methodological strengths and limitations of the studies included. Two of the included papers with “Yes” (criteria met) as a response to all the questions provided in the JBI critical appraisal checklist, while three of the included papers have one or more “Unclear”.

**Table 2:** Critical appraisal for cross sectional studies (*n* = 4)

Author (Publication year)	Were the criteria for inclusion in the sample clearly defined?	Were the study subjects and the setting described in detail?	Was the exposure measured in a valid and reliable way?	Were objective, standard criteria used for measurement of the condition?	Were confounding factors identified?	Were strategies to deal with confounding factors stated?	Were the outcomes measured in a valid and reliable way?	Was appropriate statistical analysis used?	Overall (Include, exclude, seek further) Comment for exclude
Al-Meedani et al. [24] (2016)	Unclear	Yes	Yes	Yes	Unclear	Unclear	Yes	Yes	Include
Kato et al. [21] (2017)	Yes	Yes	Unclear	Yes	Yes	Yes	Yes	Yes	Include
Sun [23] (2020)	Yes	Yes	Yes	Yes	Yes	Yes	Yes	Yes	Include
Schüler et al. [20] (2020)	Yes	Yes	Yes	Yes	Yes	Yes	Yes	Yes	Include

**Table 3:** Critical appraisal for cohort studies (*n* = 1)

Author (Publication year)	Were the two groups similar and recruited from the same population?	Were the exposures measured similarly to assign people to both exposed and unexposed groups?	Was the exposure measured in a valid and reliable way?	Were confounding factors identified?	Were strategies to deal with confounding factors stated?	Were the groups/ participants free of the outcome at the start of the study (or at the moment of exposure)?	Were the outcomes measured in a valid and reliable way?	Was the follow up time reported and sufficient to be long enough for outcomes to occur?	Was follow up complete, and if not, were the reasons to loss to follow up described and explored?	Were strategies to address incomplete follow up utilized?	Was appropriate statistical analysis used?	Overall appraisal:
Shen et al. [22] (2020)	Yes	Yes	Yes	Yes	Yes	Yes	Yes	Unclear	Yes	Yes	Yes	Include

Among the recruited five publications, four of them were performed in Asia: two in China [22, 23], one in Japan [21], and one in Saudi Arabia [24]; one further study occurred in Europe (Germany) [20]. Four of the studies were cross-sectional in design and one was longitudinal. It should be noted that participants in Schüler 's study [20] were not classified into groups based on the outcome, but on one of the predictors (preterm or full-term infants). Thus, we classified this study as a cross-sectional study instead of a case–control study as published. All the studies used questionnaires and dental or oral examinations as data-collection methods. The rank of GII of the included studies ranged from 0.084 (Germany) to 0.252 (Saudi Arabia), indicating the least and most gender-dispersed countries, respectively.

**Fig. 3:**
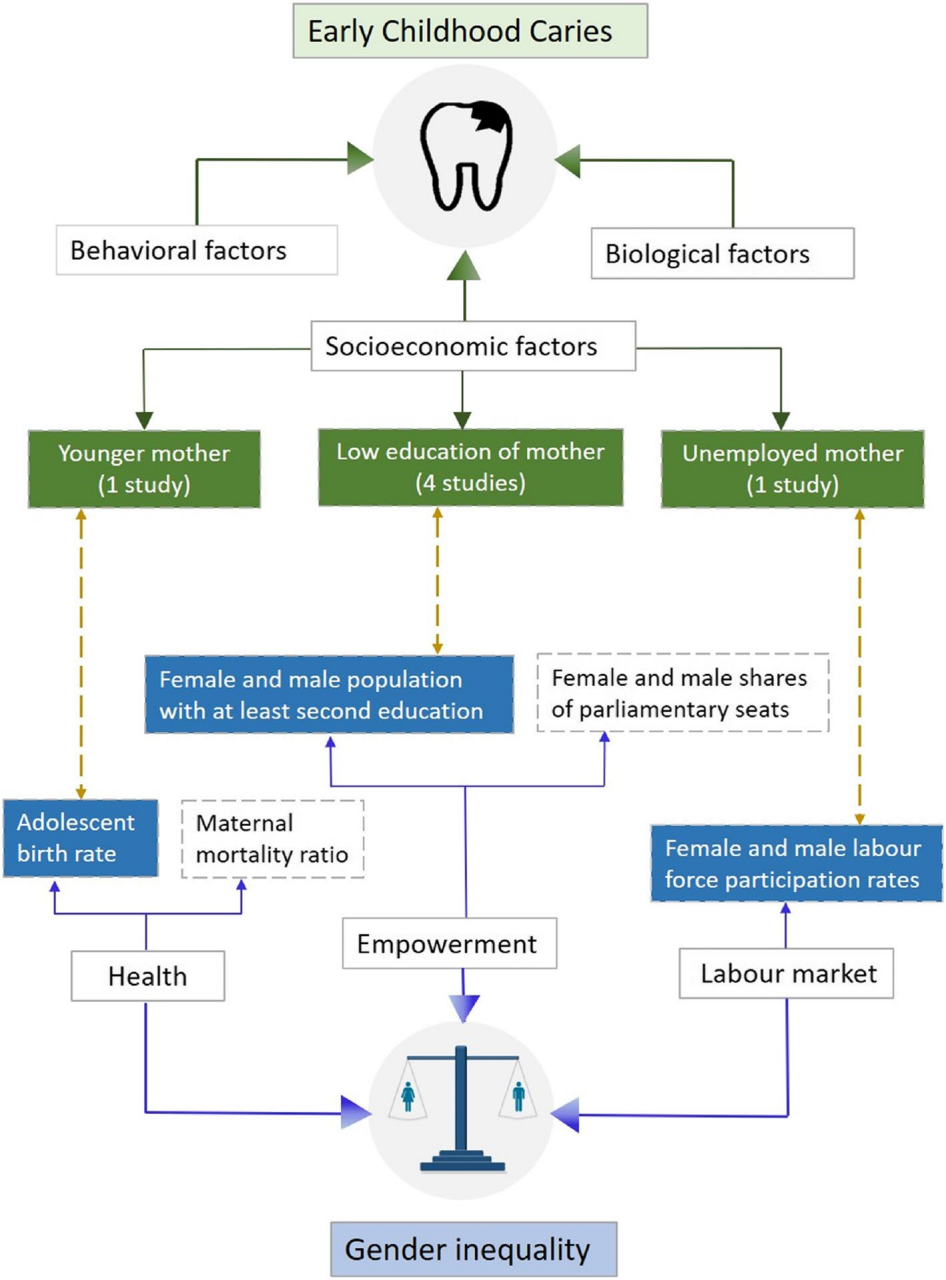
The potential association between ECC and gender inequality

Figure 3 presents the mapping of the included studies and their relationship to gender inequality. Based on the main findings regarding maternal factors associated with ECC, the studies were categorized into the following three classes: mother’s age, mother’s education level, and mother’s employment status. One of the studies found that more than one of the categorized maternal factors was associated with ECC [21]. Children of older mothers at delivery exhibited a lower risk of developing ECC [20]. Higher maternal education was associated with both lower [21, 22, 24] and higher dmft (decayed, missing and filled primary teeth) scores in children [23]. Children whose mothers were housewives or were unemployed demonstrated a higher prevalence of ECC [21].

## Discussion

Based on the findings of this scoping review, there is currently limited evidence showing an association between gender inequality and ECC at the individual level. This review also mapped evidence regarding the potential association between maternal factors related to gender inequality and ECC. The main findings indicate that maternal education level, age, and employment status are significantly associated with ECC prevalence.

In this review, we included publications limited to the recent 10 years (2012–2022), as the norms of gender inequality have changed over time worldwide [25]. We searched 425 related papers, and it was expected that many related full papers would be included. However, only five of them were recruited. Most of the included studies that reported mother-related factors associated with the ECC failed to comment that these "mother-related factors" were related to gender inequality.

Regarding the quality assessment of the included studies, the JBI critical appraisal was used as it is one of the most commonly used assessment tools to measure the methodological quality of publications [26]. It can be used to assess different study type, including cross-sectional and cohort-studies. Results from the JBI critical appraisal of this review reflected the methodological limitations of some included studies. The lack or unclear of the inclusion criteria [24], the reliability and validity of assessing the exposures [21], addressing the confounding issues [24], and the follow-up time [22] were the possible risks of bias of the included studies.

The outcome of this scoping review presented evidence that higher maternal education is associated with a decrease in ECC prevalence. Al-Meedani et al. suggested that this was due to increased awareness of health-related problems and improved oral-health practices among highly educated mothers [24]. Mothers with better dental knowledge have children with lower ECC prevalence [24, 27]. Though the current scoping review presents the possibility of maternal education as an important factor in ECC prevalence, it is unclear whether maternal education level is related to gender inequality. None of the studies included in this review addressed the difference between males and females with respect to education accessibility. However, gender inequality can affect health, as those with higher education levels can earn more and utilize benefits that improve health, such as health insurance, compared to those who are less educated [28]. An increase in educational level benefits the health of both the individual and their children [29]. Delayed access of mothers to health and oral-care services may also be linked to gender inequalities. However, little is known about the direct impact of GII and women’s access to education on the oral health of infants, toddlers, and preschool children.

The maturity of mothers may be associated with ECC prevalence in their children. Younger women are also more at risk for gender-based violence [30], which has been associated with increased risk for ECC [31]. Younger mothers are also at risk of mental health challenges and parental stress [32], both of which are also associated with an increased risk for ECC [33]. More research is needed to evaluate a direct link between harmful gender norms, adolescent pregnancy, and maternal access to education and risks of ECC.

The GII is an index measuring the equality disparities between men and women [18]. In fact, there are different indicators to measure gender inequality such as Gender-related Development Index (GDI) and Gender Empowerment Measure (GEM) which were introduced in 1995. In the current review, we adopted GII as a guidance as it is a recognised index introduced by the United Nations Development Programme in 2010 to remedy of the shortcoming of the previous GDI and GEM indices. The rank of GII of different countries based on study location is shown in Table 1; this GII was generated in 2019 with a list of 189 countries [19]. Our review included studies from countries with a wide range of GII from the lowest (Germany) to the highest (Saudi-Arabia). Besides the possibility of the gender issues, the effect of access to care on women's health and ECC status may be influenced by the states of the healthcare systems in different countries. It should also be noted that an association between GII and ECC in the ecological study cannot be extrapolated to individuals in the absence of any actual link between them.

The current study only included three factors (maternal age, education level, and employment status) that are part of the GII measurement components. Although the results of this review were unable to explore this specific theme, it should be noted that the lack of empirical studies does not rule out the possibility of a link. The relationship between gender inequality and ECC appears to be complex, and a better understanding of the interrelationships between child oral health and gender inequality is required. Based on the results of this rigorous review, conclusions regarding a direct association between gender inequality and ECC are unable to be drawn using the GII due to differences in study design, populations, sample sizes, and data-collection methods. Nevertheless, the measurement components of the GII can guide the choice of factors to explore to gain a more comprehensive understanding of this topic. Future studies comparing individual components of the GII with ECC will be required to draw conclusions regarding an association between gender inequality and ECC.

Although a scoping review seeks to present an overview of a potentially large and diverse body of literature on a broad topic. However, it still requires rigorous and transparent methods in their conduct. Therefore, we selected three prominent databases that were the most adopted in dentistry and science to ensure that the results were trustworthy. The strength of this review was that we incorporated the critical appraisal in complement with the detailed description of the included studies. The potential limitation may be that grey literatures were not searched. Although including grey literature may reduce publication bias, it may increase another risk of bias due to the uncertain quality of the publications or evidence. Due to the search strategy focused specifically on gender inequality, some ECC studies investigating maternal age, education level, and employment status may not have been represented in the search performed in this review. Notably, links between gender and ECC (for example, the prevalence of ECC in boys vs. girls) were not covered following the index of gender inequality.

In addition, we only included cross-sectional and longitudinal studies. Ecological studies and reviews that may have been subject to limitations and biases were not included, which may limit the results of this scoping review. Nevertheless, our findings suggest the plausibility of the associations between ECC and gender inequality through complex pathways that are yet to be further investigated. Future studies exploring relationships between ECC and maternal reproductive health, employment status, and political empowerment are needed to gain a better understanding of this topic and bridge this knowledge gap.

## Conclusion

There is currently limited evidence showing an association between gender inequality and ECC.

## Data Availability

All data generated or analysed during this study are included in this published article.

## Abbreviation

ECC: Early childhood caries
GII: Gender inequality index
dmft: Decayed, missing and filled primary teeth
PTI: Preterm infants
